# A Matter of Balance: Older Adults Taking Control of Falls by Building Confidence

**DOI:** 10.3389/fpubh.2014.00274

**Published:** 2015-04-27

**Authors:** Margaret Haynes, Patricia League, Gloria Neault

**Affiliations:** ^1^MaineHealth, Elder Care Services, Portland, ME, USA

**Keywords:** fall prevention program, cognitive-behavioral therapy, fear of falling, cost savings, lay leader

This commentary will present the challenges and successes of implementing and sustaining an evidence-based falls prevention program using a lay leader model. The evolution from professional educator to lay leader will be described, as well as the benefits of this model for individual participants, reducing falls and financial savings for CMS. Falls are the leading cause of death from injury and the most common cause of non-fatal injuries, resulting in emergency department visits in the older adult population with an estimated cost of over $30 billion for direct medical costs alone. Older adults who fall and are not injured may develop a fear of falling and limit activities with subsequent further loss in physical function, resulting in an increased risk of future fall (1).

A matter of balance (MOB) was developed and tested in the 1990s at Boston University’s Roybal Center for enhancement of late-life function as a comprehensive approach to maximizing activity engagement and function and reducing fall risks with funding from the National Institute on Aging (2, 3). Professionally led, utilizing physical therapists, occupational therapists, registered nurses, and social workers, this evidence-based, small group health promotion program for older adults used cognitive-behavioral techniques to reduce the fear of falling (2, 3). Participant outcomes from the randomized clinical trial (RCT) included significant improvements in falls management, falls self-efficacy, falls control, increased activity levels, and reductions in social isolation (2).

From a community perspective, utilization of health care professionals as class leads made the intervention expensive and difficult to sustain. A matter of balance/volunteer lay leader (MOB/VLL) model was developed with a translational research grant from the U.S. Administration on Aging to increase adoption of the program and thereby reach significant numbers of older adults in the community. The core elements of A MOB include (a) cognitive restructuring and behavioral activation activities that promote the belief that falls and fear of falling are controllable; (b) enhancing falls self-efficacy and falls management by helping participants set realistic goals for increasing activity; (c) promoting changes in modifiable risk factors such as securing loose rugs in their home environment; and (d) teaching exercises known to reduce risk of falling by increasing strength and balance (4). MOB/VLL maintains these cognitive restructuring activities. Experts in exercise were consulted concerning adaptations to ensure that exercises taught in the translation promoted increased strength and balance needed to reduce risk of falling and were safe for persons with osteoporosis and/or joint replacements.

Utilizing a train-the-trainer model, the partnership for healthy aging (PFHA) adapted the program, remaining true to the original MOB model. Since 2006, over 900 Master Trainers have been educated in 38 states by the PFHA in all aspects of the program utilizing a Master Trainer curriculum. Master Trainers then teach VLL utilizing a VLL curriculum and manual. A Guest Therapist handbook was developed to include a professional visit to one class to address participant concerns, demonstrate how to get up from a fall and other clinical issues. Each participant receives a participant workbook for their use at home. In the past 7 years, the translation to a lay leader model has made MOB/VLL available to over 45,000 older adults across the U.S.

Participants report significant increases in falls efficacy, falls management, and falls control at 6 weeks, 6, and 12 months, achieving comparable outcomes with those of participants in the RCT (5). The success with MOB/VLL suggests that other evidence-based programs currently requiring professional staff can be adapted for facilitation by volunteers. Further, this successful translation of a professionally led health promotion program into a volunteer lay leader model promotes embedding the program in community-based organizations, thus, making it more broadly available to older adults in diverse settings.

Volunteer lay leaders who facilitate the program report a sense of confidence about teaching, find it a rewarding experience and are enthusiastic about seeing older adults gain more independence. In a follow-up survey, lay leaders indicated that they gained a sense of accomplishment (80%), found their purpose in life had increased (48%), felt they could make a positive difference in another person’s life (76%), and increased their own confidence about managing falls (84%) (5).

The Centers for Medicare & Medicaid Services’ Evaluation of Community-based Wellness and Prevention Programs under Section 4202 (b) of the Affordable Care Act documents the economic value of MOB/VLL (6). Participation was associated with total medical cost savings, reflecting cost savings in the unplanned in-patient, skilled nursing facility, and home health settings. For example, there was a $938 decrease in total medical costs per year driven by a $517 reduction in unplanned hospitalization costs, a $234 reduction in skilled nursing facility costs, and an $81 reduction in home health costs (6).

Matter of balance/volunteer lay leader offers participating organizations the opportunity to bring an evidence-based fall prevention program to the community. A host of delivery organizations are used, including aging service providers, health departments, trauma departments, rehabilitation teams, universities, and housing. Benefits of offering an evidence-based program include new collaborations and strengthening current partnerships. It also serves as a link to support older adults living independently in the community. Creating dynamic partnerships makes this program available to numerous older adults, resulting in decreased falls, increased cost savings, and provision of continued involvement in life.

It is imperative that a MOB continues to reach older adults in the community. Strength, balance, and decreased fear of falling improve older adults’ quality of life and independence. To accomplish this, we must engage health care providers to increase referrals, enabling older adults to continue to live independently in their homes, senior housing, senior living, or assisted living. A MOB is one step for an older adult to stay engaged, but it is essential that programs are readily available and accessible.

## Conflict of Interest Statement

The authors declare that the research was conducted in the absence of any commercial or financial relationships that could be construed as a potential conflict of interest.

This paper is included in the Research Topic, “Evidence-Based Programming for Older Adults.” This Research Topic received partial funding from multiple government and private organizations/agencies; however, the views, findings, and conclusions in these articles are those of the authors and do not necessarily represent the official position of these organizations/agencies. All papers published in the Research Topic received peer review from members of the Frontiers in Public Health (Public Health Education and Promotion section) panel of Review Editors. Because this Research Topic represents work closely associated with a nationwide evidence-based movement in the US, many of the authors and/or Review Editors may have worked together previously in some fashion. Review Editors were purposively selected based on their expertise with evaluation and/or evidence-based programming for older adults. Review Editors were independent of named authors on any given article published in this volume.
